# Cervical Necrotizing Fasciitis Across the COVID-19 Pandemic: A Single-Center Exploratory Retrospective Cohort Study

**DOI:** 10.3390/jcm15145350

**Published:** 2026-07-08

**Authors:** Marko Tarle, Marina Raguž, Igor Čvrljević, Koraljka Hat, Ivica Lukšić

**Affiliations:** 1 Department of Maxillofacial and Oral Surgery, Dubrava University Hospital, 10000 Zagreb, Croatia; mtarle@kbd.hr (M.T.); igor.cvrljevic@gmail.com (I.Č.); koraljkahat@gmail.com (K.H.); 2 School of Dental Medicine, University of Zagreb, 10000 Zagreb, Croatia; 3 Department of Neurosurgery, Dubrava University Hospital, 10000 Zagreb, Croatia; marinaraguz@gmail.com; 4 School of Medicine, Catholic University of Croatia, 10000 Zagreb, Croatia; 5 School of Medicine, University of Zagreb, 10000 Zagreb, Croatia

**Keywords:** cervical necrotizing fasciitis, COVID-19 pandemic, incidence rate ratio, NPAR, CAR/PNI, odontogenic infection

## Abstract

**Background/Objectives**: Cervical necrotizing fasciitis (CNF) is a rare but rapidly progressive deep-neck infection. Reports suggest a rising incidence after the COVID-19 pandemic, but host-response biomarkers remain poorly characterized. We evaluated whether the pandemic/post-pandemic era was associated with higher CNF frequency and a distinct immunonutritional profile. **Methods**: Retrospective cohort at a Croatian national tertiary referral center. Adults with CNF were grouped by admission era: pre-pandemic (2010–2019; *n* = 11; 10.00 years) and pandemic/post-pandemic (January 2021–April 2026; *n* = 15; 5.29 years). Incidence was compared by Poisson rate ratio (IRR). Admission biomarkers included classical indices (NLR, PLR, LMR, SII, AISI) and albumin-integrated composites (CAR, NAR, NPAR, PNI, CALLY, CAR/PNI, SIRI/albumin, AISI/albumin). Mann–Whitney U and Fisher’s exact tests were used with the Benjamini–Hochberg FDR correction; ROC analysis with bootstrap 95% CIs assessed era discrimination. **Results**: Incidence rose 2.6-fold (IRR = 2.58; 95% CI 1.18–5.61; *p* = 0.021); sensitivity analysis excluding 2026 was consistent (IRR = 2.36; *p* = 0.048). All 21 odontogenic cases originated from mandibular culprit teeth. Comorbidities (27% vs. 67%) and complications (27% vs. 53%) were more frequent in the pandemic/post-pandemic era, while overall mortality was 11.5%. Among the evaluated biomarkers, NPAR (q = 0.030) and CAR/PNI (q = 0.030) showed the strongest discriminatory performance for era classification (AUC 0.867 and 0.855, respectively) within this exploratory cohort. Streptococci fell from 82% to 33% (*p* = 0.021); *S. pyogenes* disappeared, *S. anginosus*/*constellatus* persisted, and atypical organisms (*Acinetobacter*, *Candida*, *Corynebacterium*) emerged exclusively post-pandemic (0% vs. 20%). **Conclusions**: Pandemic/post-pandemic CNF was more frequent, more comorbid, and biologically distinct, with a coherent hypoalbuminemic hyperinflammatory profile with diversified microbiology. In this single-center cohort, these associations are exploratory and hypothesis-generating, and the era label denotes a calendar period rather than a confirmed individual SARS-CoV-2 infection. NPAR and CAR/PNI emerged as the most promising exploratory biomarkers and warrant prospective multicenter validation.

## 1. Introduction

Cervical necrotizing fasciitis (CNF) is an uncommon but life-threatening infection of the deep cervical fascial planes whose clinical relevance is disproportionate to its rarity. Delayed recognition can lead to airway compromise, descending mediastinitis (DNM), septic shock, carotid involvement, and death, and most published studies report mortality between 10% and 40% [1,2,3]. Successful treatment requires early imaging, urgent broad-spectrum antimicrobial coverage, source control, and aggressive surgical exploration, drainage, and debridement [1,4,5]. In our institutional practice, management is deliberately surgical and airway-focused, using multiple cervical incisions with wide decompression of the deep fascial spaces and drainage of necrotic collections, eradication of any odontogenic focus, repeated wound irrigation and surgical revision when indicated, and a low threshold for tracheotomy to secure the airway.

The growing international literature has documented changes in the epidemiology of necrotizing soft tissue infections (NSTIs) during and after the COVID-19 pandemic. These reports span anatomical sites (Fournier’s gangrene, extremity NSTI, head-and-neck necrotizing fasciitis—NF) and healthcare systems (Germany, Australia, Canada, United States, Belgium) [6,7,8,9,10,11,12,13]. A Nürnberg burns-center series reported 21, 30, and 17 NF cases in 2021, 2022, and the first five months of 2023 alone, with 94% ICU admission and 24% mortality in the 2023 sub-cohort [7]. A German level-1 trauma center analyzing 165 severe NF cases documented a marked post-pandemic excess, particularly of group A streptococcal NF [11]. The Ottawa otolaryngology group found that 68.7% of 16 consecutive head-and-neck NF cases over eight years had presented after pandemic onset [9].

Beyond case counts, a clinically useful question is whether the patients treated after the pandemic show a different host-response phenotype and whether simple admission laboratory values can capture it. Albumin, C-reactive protein (CRP), neutrophil, lymphocyte, monocyte, and platelet counts are inexpensive, routinely available variables that capture biologically distinct axes: acute inflammation, neutrophil-dominant innate activation, lymphocyte suppression, and nutritional or vascular-leak status. Classical composite indices such as the neutrophil-to-lymphocyte ratio (NLR), platelet-to-lymphocyte ratio (PLR), lymphocyte-to-monocyte ratio (LMR), systemic immune–inflammation index (SII), and aggregate index of systemic inflammation (AISI) have been developed and validated primarily in chronic oncological and cardiovascular cohorts [14,15]. More recently, a generation of composite markers that integrate inflammation with nutritional reserves has gained traction, including the CRP-to-albumin ratio (CAR), neutrophil-to-albumin ratio (NAR), neutrophil percentage-to-albumin ratio (NPAR), prognostic nutritional index (PNI), CRP–albumin–lymphocyte index (CALLY), systemic inflammation response index (SIRI), and modified Glasgow Prognostic Score (mGPS) [16,17,18,19,20]. The ratios CAR/PNI, SIRI/albumin, and AISI/albumin combine an inflammatory signal with a nutritional denominator. Together, this panel provides a compact yet multidimensional representation of the immunonutritional state. To our knowledge, it has never been applied systematically to CNF.

The biological rationale for expecting a shifted immunonutritional phenotype in the pandemic/post-pandemic era is plausible. SARS-CoV-2 infection induces marked neutrophilia with expansion of granulocytic myeloid-derived suppressor cells, concurrent T-cell lymphopenia driven by cytokine-mediated redistribution and apoptosis, and T-cell exhaustion mediated by IL-6/STAT3 signaling and PD-1/Tim-3 upregulation [21,22,23]. Importantly, these alterations do not fully resolve on clinical recovery: approximately 7–12% of moderate-to-severe COVID-19 survivors retain persistent lymphocyte subset abnormalities fifty days after symptom onset, and convalescent cohorts demonstrate elevated TNF-α/IL-10 ratios consistent with smoldering immune consumption [24,25,26]. Mania et al. have directly linked preceding or coexisting viral infection to invasive group A streptococcal disease in the post-pandemic period [27]. Parallel drivers—disrupted dental and medical care, changes in antibiotic exposure patterns, and shifts in bacterial colonization following altered social mixing—are expected to compound the biological signal. Whether these post-pandemic immunological alterations translate into a measurable host-response phenotype in CNF remains unknown.

We therefore hypothesized that CNF treated during the pandemic/post-pandemic era would show increased annualized institutional case frequency and a distinct immunonutritional inflammatory phenotype compared with the pre-pandemic era, and that simple albumin-integrated composite biomarkers would outperform classical leukocyte-only indices in capturing this phenotype. The study was designed as an observational, retrospective, era-comparison analysis; it was not designed to establish individual-level SARS-CoV-2 causation.

## 2. Materials and Methods

### 2.1. Study Design, Setting, and Era Definition

This was a single-center, retrospective, observational cohort study conducted at the Dubrava University Hospital, a large Croatian national tertiary referral center for maxillofacial and head-and-neck surgery managing severe odontogenic, oropharyngeal, and cervicofacial infections from a broad national catchment. The cohort included all consecutive adult patients treated for CNF between 1 January 2010 and 18 April 2026. Patients were grouped by admission year into a pre-pandemic era (2010–2019) and a pandemic/post-pandemic era (2021–2026). The label “pandemic/post-pandemic” was chosen deliberately to denote the healthcare and biological time period beginning with the COVID-19 pandemic and extending into subsequent years; it does not refer to a confirmed individual COVID-19 infection. Individual SARS-CoV-2 infection history, long-COVID-19 status, and vaccination status were not consistently available in the retrospective record and were therefore not analyzed. The year 2020 was treated as a transition year; there were no new CNF admissions in 2020. The study was conducted in accordance with the Declaration of Helsinki and approved by the Institutional Ethics Committee of Dubrava University Hospital, Zagreb, Croatia (2026/0423-12, 23 April 2026).

### 2.2. Inclusion and Exclusion Criteria and Diagnostic Definition

The source population comprised all consecutive adult patients (aged ≥ 18 years) admitted to and surgically managed by our department for a severe deep neck infection between 1 January 2010 and 18 April 2026. For the purposes of this study, a severe deep neck infection was defined as an acute bacterial infection involving one or more of the deep cervical fascial spaces (submandibular, sublingual, submental, perimandibular, pterygomandibular, parapharyngeal, retropharyngeal, pretracheal, carotid-sheath, and/or anterior visceral spaces) that required emergency hospital admission, intravenous antibiotic therapy, and surgical exploration with drainage, with or without airway intervention. So defined, this source category encompassed the full clinical spectrum from Ludwig’s angina and multi-space cervical abscesses to frank necrotizing fasciitis, the last of which represents its most aggressive form and the subject of the present analysis.

From this source population, a patient was included in the CNF cohort when a diagnosis of cervical necrotizing fasciitis had been established at the time of treatment on a composite of clinical, radiological and intraoperative criteria, all of which were required: (a) a compatible severe cervical infection with rapid clinical progression and/or systemic toxicity (e.g., sepsis or septic shock, pain disproportionate to local findings, rapidly spreading cervical erythema and edema, skin discoloration, bullae or crepitus); (b) contrast-enhanced CT evidence of deep cervical fascial-plane involvement, comprising diffuse soft-tissue inflammation, fascial edema, fluid collections tracking along the fascial planes and/or fascial gas; and (c) intraoperative findings characteristic of necrotizing infection, including grayish devitalized or necrotic fascia, “dishwater” purulent discharge, absence of healthy bleeding, and loss of normal fascial resistance permitting blunt finger dissection of the fascial planes, requiring wide drainage and debridement. Where tissue was submitted, histopathological evidence of fascial necrosis with an associated inflammatory infiltrate supported or confirmed the diagnosis. Both odontogenic and non-odontogenic (tonsillar/oropharyngeal) CNFs were eligible.

A patient was excluded if any of the following applied: (a) a deep neck infection lacking necrotizing features that is, a simple deep neck space abscess, phlegmon or cellulitis without the clinical, radiological and intraoperative hallmarks of necrotizing fasciitis defined above (such non-necrotizing infections were not regarded as CNF and have been addressed separately); (b) a necrotizing soft-tissue infection of a primary non-cervical site (e.g., truncal, extremity or perineal/Fournier gangrene) or necrotizing fasciitis confined to the face or to the superficial integument without involvement of the deep cervical fascial planes; and (c) age below 18 years or clinical, operative or laboratory documentation insufficient to confirm the diagnosis with the certainty required above. Isolated descending necrotizing mediastinitis without a demonstrable cervical fascial source was not classified as CNF; conversely, mediastinitis occurring as a caudal extension of a cervical necrotizing process was retained within the cohort and recorded as a complication rather than as a separate diagnosis.

### 2.3. Data Collection and Coding

For every patient, we abstracted demographics (age, gender), admission and discharge dates, length of stay, source of infection (odontogenic, defined by a recorded culprit tooth, or non-odontogenic (tonsillar/oropharyngeal) when no culprit tooth was recorded), specific culprit tooth or teeth coded in the two-digit FDI system, comorbidities, complications, and in-hospital survival outcome. Comorbidities were coded as follows: 0 = none; 1 = diabetes mellitus; 2 = arterial hypertension; 3 = oncological disease; 4 = chronic kidney disease; 5 = urinary tract infection; and 6 = pneumonia. Entries containing more than one code were treated as multiple comorbidities. Complications were coded as follows: 0 = none; 1 = descending necrotizing mediastinitis (DNM); 2 = pneumonia; 3 = carotid rupture; and 4 = osteonecrosis. Survival was coded as follows: 0 = in-hospital death; 1 = survival.

### 2.4. Biomarker Formulae

All biomarkers were calculated from admission laboratory values. Albumin was expressed in g/L; absolute leukocyte, neutrophil, lymphocyte, monocyte, and platelet counts in ×10^9^/L; and CRP in mg/L. Classical composite indices were defined as NLR = neutrophils ÷ lymphocytes, PLR = platelets ÷ lymphocytes, LMR = lymphocytes ÷ monocytes, SII = (platelets × neutrophils) ÷ lymphocytes, AISI = (neutrophils × monocytes × platelets) ÷ lymphocytes, SIRI = (neutrophils × monocytes) ÷ lymphocytes, and dNLR = neutrophils ÷ (leukocytes − neutrophils). Albumin-integrated composite biomarkers were defined as CAR = CRP ÷ albumin; NAR = neutrophils ÷ albumin; NPAR = 100 × (neutrophils ÷ leukocytes) ÷ albumin; PNI = albumin + 5 × lymphocytes (Onodera formula); CALLY = (albumin × lymphocytes) ÷ CRP × 10; CAR/PNI, SIRI/albumin, and AISI/albumin as their respective component ratios; and CRP × NLR as a multiplicative composite. The modified Glasgow Prognostic Score (mGPS) was scored as 0 (CRP ≤ 10 mg/L and albumin ≥ 35 g/L), 1 (CRP > 10 mg/L and albumin ≥ 35 g/L), or 2 (CRP > 10 mg/L and albumin < 35 g/L). The albumin-integrated composite indices (CRP/albumin ratio, neutrophil-to-albumin ratio, neutrophil-percentage-to-albumin ratio, and the prognostic nutritional index) were pre-specified rather than data-driven: they were chosen a priori to capture two complementary biological axes that we hypothesized would differ between eras, namely the intensity of the systemic inflammatory insult and the patient’s nutritional and vascular reserve, the latter being poorly reflected by conventional leukocyte-only ratios. The complete set of composite biomarker formulae, together with their component variables and primary biological axis, is summarized in Supplementary Table S1.

### 2.5. Microbiological Classification

Intraoperative deep-tissue swabs or aspirates obtained at the first surgical debridement were submitted for aerobic culture with antibiogram. Isolates were classified into the following categories: streptococci (any Streptococcus spp.); *S. pyogenes* (Group A Streptococcus)—the classical toxin-mediated “Type II” NF pathogen, reported separately; *S. anginosus/constellatus/intermedius* group (the so-called “*S. anginosus* group”, SAG)—oral/oropharyngeal commensal viridans *streptococci* recognized for slower growth in vitro, a tendency to form deep-seated abscesses, and a more indolent but progressively invasive course than *S. pyogenes*; other viridans streptococci (*S. mitis*, *S. oralis*, *S. thermophilus*); *staphylococci* (including coagulase-negative strains); atypical/unusual organisms (defined a priori as pathogens uncommon in typical odontogenic/cervicofacial infection: *A. baumannii*, *Candida albicans*, *C. striatum*, *Pseudomonas* spp., *Klebsiella* spp., *Enterococcus* spp.); polymicrobial infection (≥2 distinct bacterial genera in a single specimen); and unidentified/negative culture. We did not tabulate anaerobic organisms as a separate primary category because systematic anaerobic culturing was not performed uniformly across the entire observation period; where anaerobes were nonetheless identified on routine culture, they contributed to the polymicrobial category. Older records frequently reported “*Streptococcus* species” without species-level detail, whereas later records more often provided species-level identification; this evolving granularity is addressed in the interpretation.

### 2.6. Standardized Institutional Management

**Figure 1:**
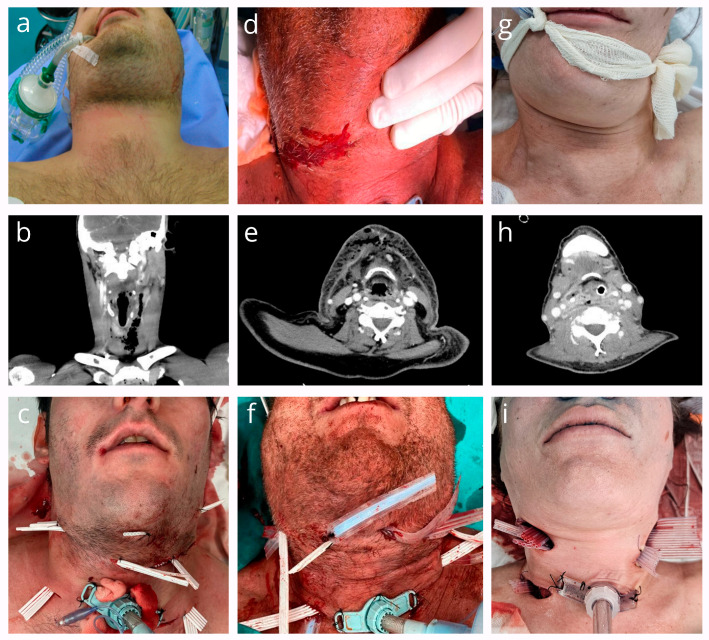
**Representative clinical and radiological presentation and surgical management of cervical necrotizing fasciitis (CNF) in three patients.** (**a**–**c**) Patient 1—a 26-year-old male with odontogenic CNF of mandibular origin (lower molar), presenting with bilateral submandibular swelling, dysphagia, and subtle anterior/right cervical erythema. (**a**) Initial clinical presentation with limited external signs despite deep infection. (**b**) Contrast-enhanced MSCT (coronal view) demonstrating extensive gas tracking within the parapharyngeal and paratracheal spaces, consistent with deep cervical fascial involvement. (**c**) Early aggressive surgical management with multiple cervical incisions (submandibular, submental, and bilateral levels II and IV) combined with tracheotomy for airway protection. (**d**–**f**) Patient 2—a 57-year-old male admitted to the intensive care unit with uncontrolled diabetes mellitus and metabolic ketoacidosis; odontogenic CNF originating from mandibular anterior teeth. (**d**) Diffuse cervical erythema and edema; upon limited initial incision, necrotic “dishwater-like” hemorrhagic fluid was encountered, confirming necrotizing infection. (**e**) Axial contrast-enhanced CT showing extensive subcutaneous emphysema in submental and submandibular regions, obliteration of normal cervical fascial planes, and diffuse fat stranding. (**f**) Definitive surgical treatment with wide cervicofacial fasciotomy via multiple incisions extending to temporal regions, along with tracheotomy. (**g**–**i**) Patient 3—a 42-year-old female admitted after 10 days of pharyngitis with odynophagia and dysphagia; non-odontogenic CNF complicated by descending mediastinitis. (**g**) Absence of significant external cervical findings despite advanced deep infection. (**h**) Contrast-enhanced axial CT demonstrating parapharyngeal and retropharyngeal fluid collections with locules of gas, indicating deep space infection with early necrotizing features. (**i**) Extensive surgical drainage via multiple cervical incisions with placement of drains into parapharyngeal and retropharyngeal spaces, combined with tracheotomy. The figure illustrates the frequent discordance between external clinical findings and the extent of deep cervical infection, the critical role of contrast-enhanced CT in early diagnosis, and the institution’s aggressive surgical strategy, including wide cervical decompression, multiple drainage incisions, and early airway control as key components of CNF management.

**Figure 2:**
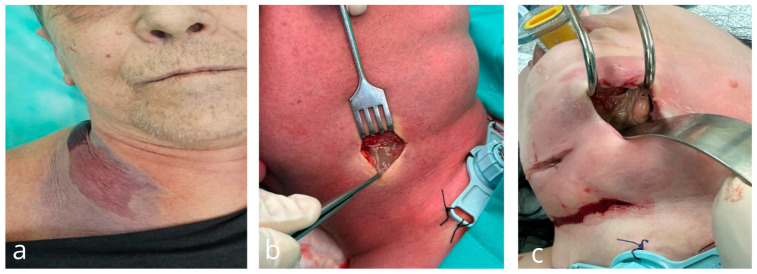
**Clinical intraoperative findings in cervical necrotizing fasciitis.** (**a**) Cutaneous manifestation of CNF with livid discoloration and skin necrosis in the lateral neck region, reflecting underlying microvascular thrombosis and tissue ischemia. (**b**) Intraoperative finding after division of the platysma demonstrating characteristic necrotic “dishwater-like” fluid, indicative of fascial necrosis and deep soft tissue infection. (**c**) Advanced tissue destruction in the submandibular region with evident necrosis of soft tissues, including involvement of the submandibular gland, confirming extensive spread along cervical fascial planes. This figure highlights the spectrum of clinical and intraoperative features of CNF, from subtle cutaneous changes to overt tissue necrosis, underscoring the importance of early surgical exploration and aggressive debridement.

Throughout both eras, all patients were managed according to a standardized institutional protocol consisting of early airway assessment and control, including tracheotomy when clinically indicated; aggressive surgical source control with multiple cervical incisions enabling wide decompression and drainage of the deep neck fascial spaces and necrotic or purulent collections; eradication of any odontogenic focus; repeated open wound irrigation and dressing changes with serial surgical revision when indicated according to clinical progression and wound appearance; broad-spectrum empirical intravenous antibiotic therapy (typically a beta-lactam/beta-lactamase inhibitor combination with metronidazole and gentamicin, escalated to meropenem and vancomycin with antifungal coverage when indicated) subsequently narrowed according to microbiological culture and antibiogram results; intensive-care monitoring with goal-directed hemodynamic, respiratory and nutritional support; and dental source management including extractions and oral sanation where indicated (Figure 1 and Figure 2). The uniformity of this surgical- and airway-focused approach across both eras minimizes the risk that observed era differences reflect changes in treatment practice.

### 2.7. Statistical Analysis

Continuous variables were summarized as the median and interquartile range (IQR) and compared using the Mann–Whitney U test. Effect size for continuous non-parametric comparisons was expressed as Cliff’s delta (δ) oriented as pandemic/post-pandemic minus pre-pandemic. Categorical variables were summarized as counts and percentages and compared using Fisher’s exact test. Annualized institutional case frequency was compared by exact Poisson rate ratio with calendar years as exposure time; the primary comparison used 10.00 pre-pandemic years (2010–2019) and 5.29 pandemic/post-pandemic years (January 2021–18 April 2026). Because 2026 represented an incomplete calendar year, a sensitivity analysis using 10.00 vs. 5.00 years and 11 vs. 13 cases was also performed. The exact conditional Poisson test was used for significance. ROC analysis with bootstrap (2000 resamples) and 95% confidence intervals for AUC assessed the ability of individual markers to discriminate the pandemic/post-pandemic era from the pre-pandemic era; optimal cut-offs were selected using Youden’s index. Exploratory age/sex-adjusted logistic regression was performed for single-marker association with era; highly collinear composite scores were not entered simultaneously with their mathematical components. Leave-one-out cross-validation (LOOCV) was used to estimate the internal discriminatory performance of candidate parsimonious models. Benjamini–Hochberg (BH) false-discovery-rate-adjusted q values were calculated for the continuous biomarker panel as exploratory multiplicity control. Two-sided *p* < 0.05 was considered statistically significant. All analyses were performed in MedCalc, version 12.5.0 (MedCalc Software, Ostend, Belgium; https://www.medcalc.org, accessed on 18 April 2026). ROC analysis distinguished the calendar era, not individual COVID-19 exposure. No a priori power calculation was performed; the sample size was fixed by the rarity of cervical necrotizing fasciitis over the available period, and all inferential analyses are therefore exploratory and hypothesis-generating. Complete blood count, CRP, and serum albumin were available for all 26 patients, so no imputation was required for the core biomarker panel; variables that were not uniformly recorded across the whole period, including the individual components required to reconstruct the LRINEC score and body mass index, were not analyzed. Procalcitonin, which entered routine use in the workup of necrotizing fasciitis at our institution only in 2020, was available for the pandemic/post-pandemic cohort but not for pre-pandemic patients; it is therefore reported descriptively for the later era only and was not used for between-era comparison. The number of outcome events was small relative to the number of candidate predictors, with only 11 events in the pre-pandemic stratum, so each logistic model was limited to a single biomarker together with age and sex. The models were not mutually adjusted, and the resulting odds ratios, confidence intervals, Youden-derived cut-offs, and leave-one-out estimates are optimism-prone and carry a real risk of overfitting. They should be regarded as internal, exploratory estimates that require external validation in an independent multicenter cohort before any inference about generalizability or clinical performance is drawn.

## 3. Results

### 3.1. Temporal Distribution and Incidence Rate Ratio

**Figure 3:**
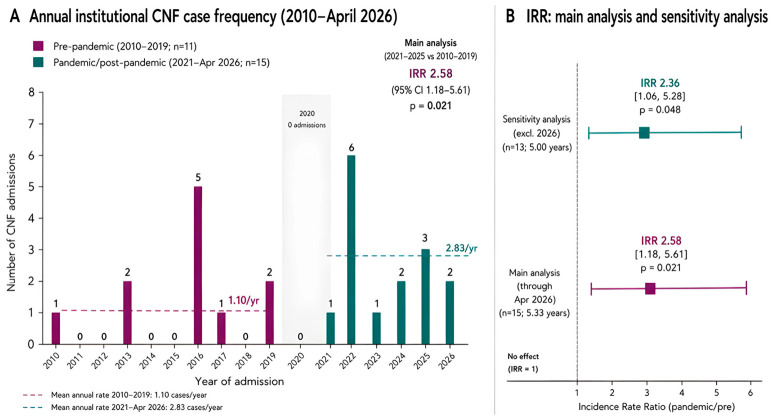
Institutional incidence of cervical necrotizing fasciitis. (**A**) Annual case counts 2010–April 2026. Gray band: transition year 2020 with zero admissions. Dashed horizontal lines: mean annual rate for each era. The IRR call-out box presents the main Poisson analysis. (**B**) Main IRR plus sensitivity analysis excluding the partial year 2026, both remaining significantly above the null (IRR = 1).

During the pre-pandemic decade (2010–2019; 10.00 observation-years), 11 CNF cases were recorded—a mean rate of 1.10 cases/year. During the pandemic/post-pandemic period (2021–April 2026; 5.29 observation-years), 15 cases were recorded—2.83 cases/year—2.6-fold higher than the pre-pandemic reference (IRR = 2.58; 95% CI 1.18–5.61; exact conditional Poisson *p* = 0.021). A sensitivity analysis excluding the partial year 2026 (13 cases in 5.00 years) yielded IRR = 2.36 (95% CI 1.06–5.28; *p* = 0.048), confirming the robustness of the signal (Figure 3). A striking feature was the complete absence of CNF admissions during 2020, followed by a sharp rebound peaking at six cases in 2022, more than any prior year of the observation period, and continued elevated activity through 2024–2026.

### 3.2. Demographics, Comorbidities, Complications, Source of Infection, and Mortality

Demographic and clinical characteristics are summarized in Table 1. Gender distribution was nearly identical across eras (male: 8/11 = 73% pre-pandemic vs. 11/15 = 73% pandemic/post-pandemic), with male predominance in both eras. Pandemic/post-pandemic patients were numerically older (median 49 vs. 39 years) and had longer hospitalizations (median 21 vs. 12 days), although neither difference reached statistical significance.

**Figure 4:**
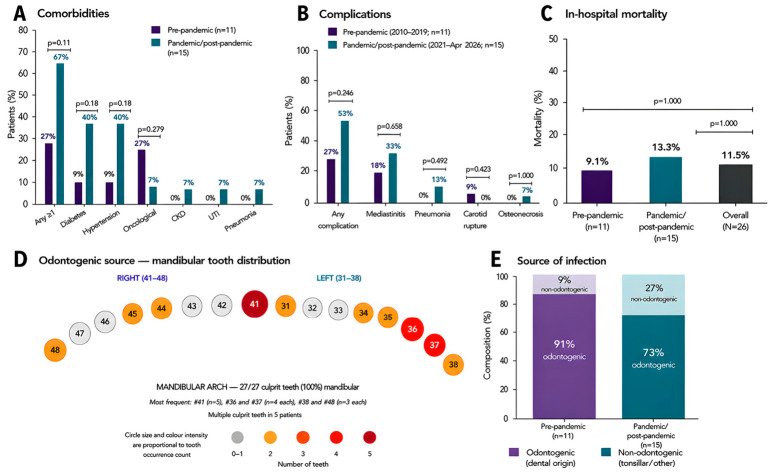
Clinical characteristics across eras. (**A**) Comorbidity prevalence. (**B**) Complication rates. (**C**) In-hospital mortality—institutional, pandemic/post-pandemic, and overall. (**D**) Dental chart of mandibular culprit teeth pooled across the 21-patient odontogenic subgroup; circle size and color intensity are proportional to tooth occurrence count. All 27 culprit teeth were mandibular. (**E**) Odontogenic vs. non-odontogenic source by era.

The burden of comorbidities increased substantially in the pandemic/post-pandemic era. The proportion of patients with at least one comorbidity rose from 3/11 (27%) to 10/15 (67%), driven by diabetes mellitus (9% vs. 40%) and arterial hypertension (9% vs. 40%). The only comorbidity category that declined was oncological disease (27% vs. 7%). The four oncological cases comprised one multiple myeloma and three head-and-neck squamous cell carcinomas (of the hypopharynx, the floor of the mouth, and the lower lip). Complications were nearly twice as frequent in the pandemic/post-pandemic era: any complication 27% vs. 53%, descending necrotizing mediastinitis 18% vs. 33%, and hospital-acquired pneumonia 0% vs. 13%. One case of carotid rupture occurred pre-pandemic and one case of mandibular osteonecrosis post-pandemic (Figure 4A,B).

Odontogenic infection was the dominant source, accounting for 21/26 (81%) of cases; the remaining 5/26 (19%) were non-odontogenic, arising from tonsillar or other oropharyngeal sources. The proportion of non-odontogenic cases was numerically higher in the pandemic/post-pandemic era (27% vs. 9%), although this did not reach statistical significance. Strikingly, all 27 recorded culprit teeth were mandibular; no maxillary teeth were involved. The most frequent culprit tooth was the lower central incisor #41 (*n* = 5), followed by the lower first and second molars #36 and #37 (*n* = 4 each) and the third molars #38 and #48 (*n* = 3 each). Molars (#6, #7, #8 positions in either mandibular quadrant) accounted for 15/27 (56%) of all culprit teeth. Five patients had multiple culprit teeth (Figure 4D,E).

Overall in-hospital mortality was 3/26 (11.5%). Mortality was 1/11 (9.1%) pre-pandemic and 2/15 (13.3%) pandemic/post-pandemic, not significantly different (*p* = 1.00). All three deceased patients were aged 59 years or older and had at least one serious complication (Figure 4C).

### 3.3. Immunonutritional Biomarker Profile Across Eras

**Figure 5:**
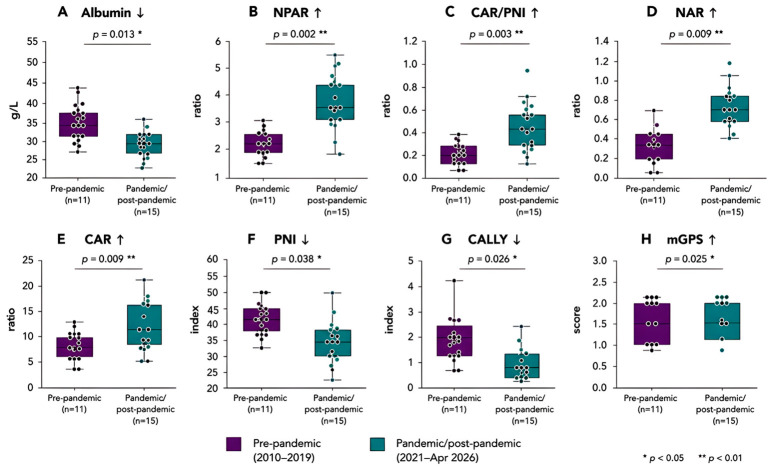
Distribution of eight key admission biomarkers across eras. Box-and-whisker plots with jittered individual data points. Significance: * *p* < 0.05, ** *p* < 0.01, NPAR was the single most discriminative marker.

**Figure 6:**
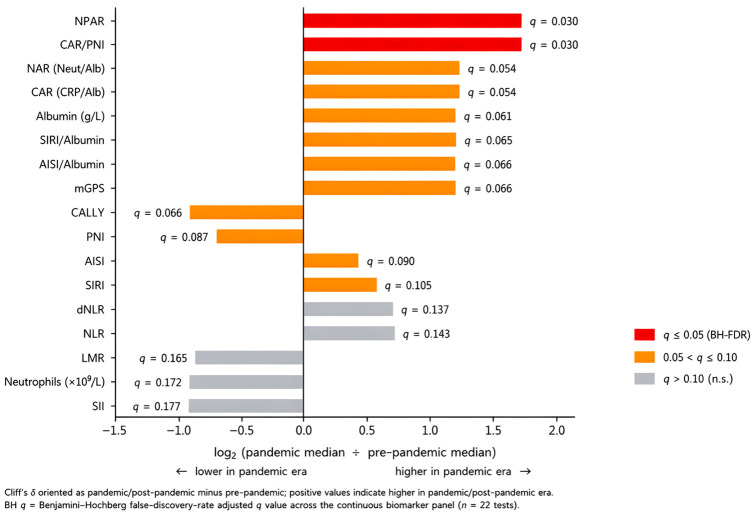
Ranked direction and strength of era differences across biomarkers. Bars represent log_2_ (pandemic median ÷ pre-pandemic median); color denotes BH-FDR significance tier according to the corresponding q value. The highest-ranked biomarkers were predominantly albumin-integrated composite markers (NPAR, CAR/PNI, CAR, NAR, albumin, and SIRI/albumin), whereas classical leukocyte-only indices generally showed weaker discriminatory performance. Only NPAR and CAR/PNI remained statistically significant after BH-FDR correction (q = 0.030 for both).

Admission laboratory values and composite biomarkers are presented in Table 2, with visual distributions for the most discriminative markers shown in Figure 5 and the global ranking of era differences in Figure 6. Among standard parameters, only serum albumin reached statistical significance, with a marked drop from a pre-pandemic median of 33.0 g/L to a pandemic/post-pandemic median of 28.0 g/L (*p* = 0.013; Cliff’s δ = −0.58). CRP, absolute neutrophils, and platelets were numerically higher, and absolute lymphocytes numerically lower, in the pandemic/post-pandemic era, directionally consistent with a more intense acute-phase response and relative lymphopenia, but none reached significance in isolation. Procalcitonin, introduced into routine use at our institution only in 2020, was available for the pandemic/post-pandemic cohort (14 of 15 patients) but not for any pre-pandemic patient; in the later-era cohort, admission procalcitonin was markedly elevated and widely dispersed (median 2.10 ng/mL, IQR 0.76–11.36; range 0.50–14.65), consistent with a severe systemic bacterial inflammatory state. Because no pre-pandemic values were available, procalcitonin was not compared between eras.

The composite biomarker panel produced a far more coherent picture. Ten markers showed statistically significant era differences, all in the biologically expected direction: NPAR (*p* = 0.002; BH-FDR q = 0.030), CAR/PNI (*p* = 0.003; q = 0.030), CAR (*p* = 0.009), NAR (*p* = 0.009), SIRI/albumin (*p* = 0.017), AISI/albumin (*p* = 0.022), mGPS (*p* = 0.025), CALLY (*p* = 0.026), PNI (*p* = 0.038), and AISI (*p* = 0.043). Three further markers showed trends in the same direction: SIRI (*p* = 0.055), CRP × NLR (*p* = 0.062), and dNLR (*p* = 0.078). After the Benjamini–Hochberg false-discovery-rate correction across the 22 tested biomarkers, only NPAR and CAR/PNI remained statistically significant (q = 0.030 for both), whereas all other biomarker associations should be regarded as exploratory and hypothesis-generating. NPAR and CAR/PNI were also the highest-ranked by effect size (Cliff’s δ = +0.73 and +0.71, respectively). The classical leukocyte-only indices (NLR, PLR, SII, LMR) all showed directionally consistent changes but failed to reach significance individually, a pattern examined in detail in the Discussion.

### 3.4. Era Discrimination: ROC Analysis and Exploratory Multivariable Models

**Figure 7:**
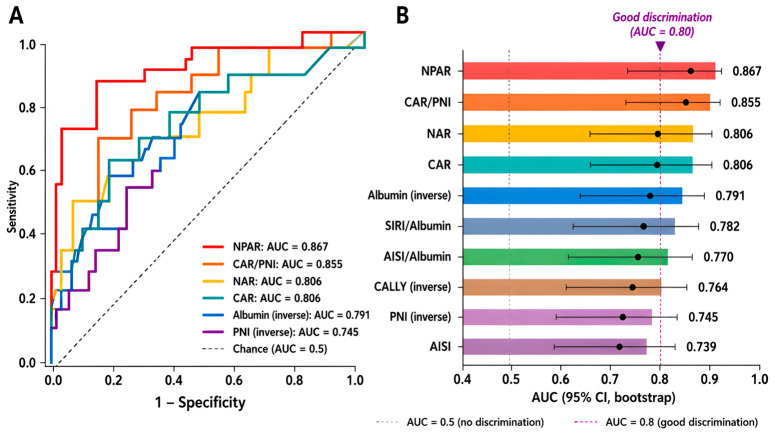
Era discrimination by individual biomarkers. (**A**) ROC curves for the six top-ranked single-variable classifiers; NPAR and CAR/PNI achieve AUCs above 0.85. (**B**) Ranked AUC values with bootstrap 95% confidence intervals across the ten leading biomarkers. The dotted line at AUC = 0.80 marks the conventional threshold for good discrimination.

To quantify how well individual biomarkers could discriminate the pandemic/post-pandemic era from the pre-pandemic era, we performed ROC analysis with bootstrap 95% confidence intervals (Figure 7; Table 3). NPAR emerged as the single strongest classifier (AUC = 0.867; 95% CI 0.697–0.994), followed closely by CAR/PNI (AUC = 0.855; 95% CI 0.691–0.976). Both indices achieved perfect specificity of 100% at the Youden-optimal cut-off (NPAR ≥ 2.79; CAR/PNI ≥ 0.283), with a sensitivity of 73%. NAR and CAR both showed good discrimination (AUC = 0.806 each), followed by albumin (AUC = 0.791), SIRI/albumin (AUC = 0.782), and AISI/albumin (AUC = 0.770). All six top-ranked ROC markers integrate a cellular inflammatory component with serum albumin, whereas classical leukocyte-only indices ranked below them. ROC analysis was interpreted as characterizing the era’s phenotype, not as a diagnostic test for individual COVID-19 exposure.

In exploratory age- and sex-adjusted logistic regression, NAR (OR = 2.85 per 0.1 unit, 95% CI 1.20–6.77, *p* = 0.018), CAR (OR = 1.09 per 5 units, 1.01–1.18, *p* = 0.028), CAR/PNI (OR = 4.45 per 0.1 unit, 1.21–16.37, *p* = 0.025), NPAR (OR = 10.94 per unit, 1.11–107.93, *p* = 0.040), and SIRI/albumin (OR = 1.54 per 0.1 unit, 1.00–2.35, *p* = 0.048) remained significantly associated with the pandemic/post-pandemic era after adjustment. In leave-one-out cross-validation of candidate parsimonious multivariable models, a two-variable albumin + AISI model achieved the highest internal cross-validated AUC (LOOCV AUC = 0.830), followed by NPAR alone (0.812) and CAR/PNI alone (0.806). Given the sample size (*n* = 26), these models should be considered exploratory and require external multicenter validation before clinical deployment.

### 3.5. Microbiological Spectrum Across Eras

**Figure 8:**
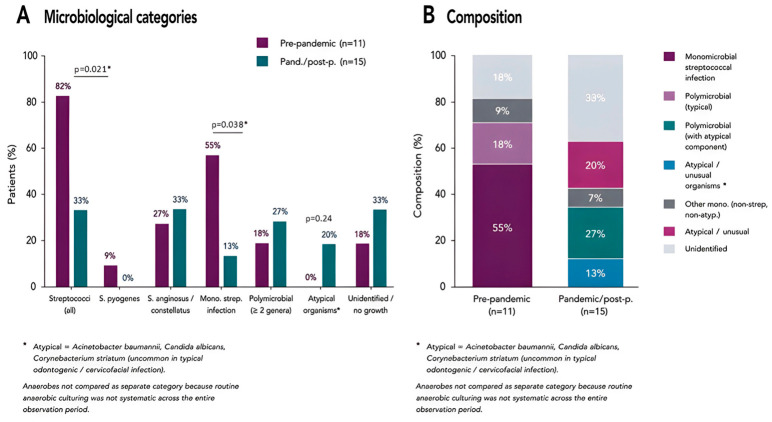
Microbiological spectrum across eras. (**A**) Proportion of patients in each microbiological category. *S. pyogenes* is lost in the pandemic/post-pandemic cohort, while *S. anginosus/constellatus* is maintained and effectively becomes the dominant streptococcal lineage. (**B**) Compositional view demonstrating the reduction in the monomicrobial-streptococcal fraction and the emergence of an atypical-organisms fraction post-pandemic, alongside a larger unidentified-culture fraction.

Microbiological findings are summarized in Table 4 and visualized in Figure 8. Two changes reached statistical significance. First, the overall proportion of patients with any streptococcal isolate fell from 9/11 (82%) pre-pandemic to 5/15 (33%) pandemic/post-pandemic (*p* = 0.021). Second, strictly monomicrobial streptococcal infection, the textbook phenotype of classical odontogenic CNF, declined from 6/11 (55%) to 2/15 (13%) (*p* = 0.038).

Within the streptococcal family, the substructure of the shift was clinically illuminating. *S. pyogenes* (Group A *streptococcus*) was isolated in one pre-pandemic patient (9%) and in zero pandemic/post-pandemic patients (0%). The *S. anginosus*/*constellatus* group, in contrast, was present in 3/11 (27%) pre-pandemic and 5/15 (33%) pandemic/post-pandemic patients, remaining the dominant streptococcal lineage after the pandemic and effectively displacing *S. pyogenes* as the characteristic streptococcal pathogen. This distinction is examined further in the Discussion; species-level data are, however, limited by evolving laboratory identification practices over time, as older records frequently reported “*Streptococcus* species” without species-level detail.

Atypical/unusual organisms, defined a priori as pathogens uncommon in typical odontogenic cervicofacial infection, were entirely absent pre-pandemic but were isolated in 3/15 (20%) pandemic/post-pandemic patients: *Corynebacterium striatum*, *Acinetobacter baumannii*, and *Candida albicans* (one each). Although the 0% vs. 20% difference did not reach statistical significance given the small sample size (*p* = 0.238), these exploratory observations, based on very small numbers, are of potential clinical interest because all three organisms are from categories with distinct treatment implications. Polymicrobial infections were numerically more frequent in the pandemic/post-pandemic period (27% vs. 18%), but more meaningfully, their internal composition changed: pre-pandemic polymicrobial infections combined *streptococci* with other common oral-flora species, whereas pandemic/post-pandemic polymicrobial infections often included an atypical component. The proportion of cases with no organism identified was higher in the pandemic/post-pandemic period (33% vs. 18%), plausibly reflecting more widespread pre-admission antibiotic exposure rather than a true biological phenomenon.

## 4. Discussion

This single-center, observational study of cervical necrotizing fasciitis, conducted at a Croatian national tertiary referral center and spanning sixteen years, documents three coherent and mutually reinforcing findings that together argue for a qualitatively altered disease profile in the pandemic/post-pandemic era. First, the annualized institutional case frequency more than doubled after the pandemic onset, with an incidence rate ratio of 2.58 robust to sensitivity analysis. Second, the later-era patients were not merely more numerous: they presented with a coherent hypoalbuminemic, neutrophil-predominant, hyperinflammatory immunonutritional profile. This profile was captured with striking fidelity by simple albumin-integrated composite biomarkers, NPAR, CAR/PNI, NAR, CAR, SIRI/albumin and AISI/albumin, which showed stronger discriminatory performance than classical leukocyte-only indices (NLR, PLR, SII, LMR) in both univariate Mann–Whitney comparisons and ROC era-discrimination analysis. NPAR alone achieved an AUC of 0.867, and a parsimonious albumin + AISI combination reached a leave-one-out cross-validated AUC of 0.830. These findings should be interpreted cautiously given the small sample size and lack of external validation. Third, the microbiological landscape diversified: total streptococcal isolation halved, S. pyogenes disappeared, the S. anginosus/constellatus group persisted as the dominant streptococcal lineage, and three atypical organisms (Acinetobacter baumannii, Candida albicans, Corynebacterium striatum) exclusively appeared in the later era. Although exploratory, these epidemiological, immunonutritional, and microbiological observations were directionally concordant and may together suggest a broader shift in the institutional CNF profile during the pandemic/post-pandemic era. The magnitude of our incidence rise is fully consistent with the growing international literature documenting increased necrotizing soft-tissue infections after the pandemic across anatomical sites and healthcare systems [6,7,8,9,10,11,12], and our Croatian CNF data provide the first formal Poisson IRR estimate applied specifically to head-and-neck CNF. The complete absence of CNF admissions to our institution during 2020 should be interpreted with caution. Our hospital functioned as a designated COVID-19 facility during the first pandemic wave, with substantial reorganization of non-COVID-19 services, which may have temporarily altered referral patterns for severe cervicofacial infections. We cannot exclude that some CNF cases occurring nationally during 2020 were managed at alternative regional centers rather than reflecting a true biological absence. Nevertheless, the marked rebound observed in 2021–2022, with six cases in 2022 alone, exceeding any prior single year of our observation period, is consistent with a deferred-presentation pattern, in which patients discouraged or unable to access dental and emergency care during lockdown harbored odontogenic or tonsillar foci that subsequently presented in larger numbers once healthcare access normalized. This pattern parallels the catch-up phenomenon documented for other time-critical surgical emergencies [28,29].

To place these institutional figures in a national context, the rarity of cervical necrotizing fasciitis is itself relevant to how this series should be read. Necrotizing fasciitis of all anatomical sites has a reported incidence of roughly 0.4 to 1.4 cases per 100,000 population per year in contemporary European and North American series [1,2], and the cervical form represents only a small fraction of this total. For a national catchment of approximately 3.9 million inhabitants, the expected number of cervical necrotizing fasciitis cases is therefore of the order of only one to a few per year nationwide. Against this background, 26 histologically and intraoperatively confirmed cases accrued at a single referral center, even across a sixteen-year window, represent a comparatively large institutional experience rather than an isolated case collection: several published head-and-neck necrotizing fasciitis cohorts that have informed current management comprise similar or smaller numbers, including the sixteen-case eight-year series from Ottawa cited above [9]. We therefore present this work as a single-center cohort study with formal time-based incidence modeling, while remaining explicit that the absolute event counts are small and that all biomarker-level inferences are correspondingly exploratory.

The immunological interpretation of the biomarker signature deserves particular emphasis because it may provide a plausible biological bridge between the pandemic and the altered CNF phenotype. Albumin was substantially lower in the pandemic/post-pandemic era (median 28.0 vs. 33.0 g/L, *p* = 0.013), while CRP/albumin, neutrophil/albumin, and neutrophil-percentage/albumin ratios were all higher. Albumin functions simultaneously as a negative acute-phase reactant, a marker of hepatic priority reallocation away from protein synthesis under systemic inflammatory stress, a quantitative measure of visceral protein reserve, and an indicator of capillary permeability and vascular leak. In severe infection, hypoalbuminemia reflects the confluence of capillary leakage, hepatic reprioritization, catabolism, and pre-existing nutritional frailty. The simultaneous trend toward higher absolute neutrophils and lower absolute lymphocytes, together with the elevation of neutrophil-dominant composite indices, is compatible with an innate-dominant, lymphocyte-suppressed inflammatory state. This pattern is compatible with, though not specific to, the hematological changes reported in acute and convalescent SARS-CoV-2 infections. COVID-19 drives neutrophilia with the expansion of granulocytic myeloid-derived suppressor cells that suppress T-cell function [21,22]; it produces T-cell lymphopenia through cytokine-mediated redistribution, apoptosis, and PD-1/Tim-3-driven exhaustion [23]; and it is accompanied by hypoalbuminemia of substantial prognostic weight [24]. Critically, these alterations do not fully resolve on clinical recovery; 7–12% of moderate-to-severe COVID-19 survivors retain persistent B-cell, CD4+ T-cell, and regulatory T-cell abnormalities fifty days after symptom onset [25], and convalescent cohorts show elevated TNF-α/IL-10 ratios indicating ongoing immune consumption even in the absence of overt long-COVID-19 symptoms [26]. In this biological landscape, an impaired host response coexisting with a conserved or enhanced pro-inflammatory potential could plausibly create conditions under which commensal or opportunistic organisms—*S. anginosus* group members, *Candida*, and MDR Gram-negatives—can escalate from latent colonization to fulminant fascial necrosis, while simultaneously producing a disproportionate systemic inflammatory response captured in our composite biomarkers. Mania et al. have directly linked preceding or coexisting viral infection to invasive group A streptococcal disease in the post-pandemic period [27], and analogous mechanisms likely extend to the oropharyngeal streptococcal spectrum. We emphasize that our cross-sectional retrospective data cannot establish individual-level causation; the COVID-19 immune-dysregulation hypothesis is advanced on the basis of biological plausibility, temporal association, and biomarker congruence, not on individual COVID-19 exposure documentation, which was not available.

A further consequence of the hypoalbuminemic phenotype deserves specific mechanistic emphasis because it links the biomarker shift directly to surgical strategy. Albumin is the principal determinant of plasma colloid osmotic pressure, and a fall in median albumin from 33 to 28 g/L meaningfully reduces the intravascular oncotic gradient. In the setting of an intense local inflammatory response, with endothelial activation and degradation of the endothelial glycocalyx, this loss of oncotic pressure promotes fluid extravasation into the loose areolar tissue of the deep cervical fascial planes [30]. In the confined yet highly compliant spaces of the neck, the resulting interstitial edema can spread rapidly along fascial planes and encroach on the laryngopharyngeal airway, converting a deep-space infection into an immediate airway emergency. This chain, in which hypoalbuminemia lowers colloid osmotic pressure and, together with inflammatory capillary leak, accelerates cervical and laryngopharyngeal edema, offers a coherent explanation for why an immunonutritional shift toward lower albumin may translate into clinically more aggressive disease, and it reinforces our institutional emphasis on early airway assessment and securement alongside wide surgical debridement in this patient group. We note, however, that airway interventions and edema severity were not systematically quantified in this retrospective record, so this pathway remains a biologically grounded hypothesis rather than a demonstrated mediation.

A notable negative finding is that most classical leukocyte-only indices (NLR, PLR, SII, LMR) did not reach significance in our cohort, even though the pandemic/post-pandemic CNF cases were clearly more severe by nearly every other metric. This is not peculiar to our sample but a recognized pattern. Classical indices were derived and validated primarily in chronic oncological and cardiovascular cohorts [14,15], where the relationship between circulating cell distributions and prognosis unfolds over weeks to months. In acute severe surgical infection, the inflammatory response is rapid, saturated, and dominated by absolute neutrophilia, so relative ratios plateau or become erratic, and their discriminatory value collapses. A recent systematic evaluation of NSTI predictive scores concluded that NLR and PLR lack specificity for NSTI and that further research is needed to define useful cut-offs [31]. The albumin-integrated markers that performed well in our data (NPAR, CAR/PNI, NAR, CAR) share a common architecture: they multiply or divide an inflammatory signal by serum albumin or lymphocyte count. They therefore capture two physiologically distinct dimensions simultaneously: the magnitude of the inflammatory hit and the host’s nutritional and vascular-integrity reserve to absorb it. This dual nature is exactly what an acute catabolic condition like CNF requires, and it is consistent with our observation that the six top-ranked markers in the ROC analysis all combine inflammation with albumin, whereas leukocyte-only indices cluster below. This suggests that albumin is not a passive covariate in CNF but a central marker of host vulnerability, and that future risk-stratification work should incorporate it explicitly. Importantly, after the Benjamini–Hochberg false-discovery-rate correction, only NPAR and CAR/PNI retained statistical significance, whereas the remaining biomarker associations should be regarded as exploratory and hypothesis-generating. Because all top-ranked composites share albumin as a component and their confidence intervals overlap that of albumin alone, we cannot claim statistical superiority of any single index over serum albumin in this small sample; the albumin-integrated indices are best regarded as convenient combined summaries of the inflammation–nutrition axis, to be compared formally in larger cohorts.

The microbiological findings deserve careful framing. The near-complete loss of S. pyogenes and continued predominance of the *S. anginosus* group offer a parsimonious biological interpretation that dovetails with the immunological data. *S. pyogenes* is a classical toxin-driven, fast-replicating “Type II” pathogen whose severe disease manifestations typically require exposure of an immunologically naive or only lightly primed host. The *S. anginosus* group (SAG), comprising *S. anginosus*, *S. constellatus,* and *S. intermedius*, consists of slow-growing oral and oropharyngeal commensal-like organisms that are generally harmless in healthy hosts but are recognized in the microbiological literature for producing deep-seated abscesses and progressively invasive suppurative infections [32,33]. In hosts with reduced T-cell function, altered neutrophil biology, and depleted albumin reserves, i.e., the hosts emerging from the pandemic, pathogens that would previously have been contained at the mucosal level can escalate into the fascial planes and produce CNF. The simultaneous emergence of *Candida* and MDR Gram-negative commensals (*A. baumannii, C. striatum*) fits the same template: opportunistic organisms exploiting a depressed host. We acknowledge three limitations that temper this interpretation. First, routine anaerobic culturing was not uniform across our observation period, so anaerobes cannot be compared as a distinct primary category. Second, species-level streptococcal reporting evolved over time; older records more often contained only “*Streptococcus* species”, so part of the apparent shift toward SAG may reflect improved microbiological identification rather than a pure biological change. Third, the apparent increase in unidentified cultures post-pandemic likely reflects more widespread community antibiotic exposure during and after the pandemic [34]. Even accounting for these methodological caveats, the disappearance of *S. pyogenes* and the emergence of three atypical organisms remain robust observations. Because no formal antibiogram analysis was performed, these observations should not be interpreted as evidence of changing antimicrobial susceptibility patterns. The emergence of atypical and fungal organisms may justify future evaluation of empirical antimicrobial strategies in larger cohorts, but any implications for antibiotic selection remain hypothesis-generating.

The anatomical distribution of odontogenic sources deserves comment. Odontogenic infection accounted for 81% of cases in our cohort, and remarkably, all 27 recorded culprit teeth were mandibular, with no maxillary involvement. The most frequent culprit teeth were the lower central incisor (#41), followed by the lower first and second molars (#36, #37) and the third molars (#38, #48); molars overall represented 56% of culprit teeth. This pattern mirrors the known anatomy of mandibular odontogenic infections, which drain through the submandibular, sublingual, and submental spaces into the deeper cervical fascial compartments, enabling rapid cervical spread once mucosal barriers are breached [5,35]. The concentration of culprits in the mandibular frontal segment and molar region reflects both the anatomical proximity of these tooth apices to the lingual cortex (allowing lingual perforation into the submandibular space) and the epidemiology of periapical and pericoronal infections in those locations. Five patients had multiple culprit teeth, perhaps reflecting more advanced dental disease or delayed dental treatment in some cases. The tendency toward more non-odontogenic (tonsillar/oropharyngeal) sources in the pandemic/post-pandemic era (27% vs. 9%) is consistent with the documented post-pandemic increase in severe oropharyngeal streptococcal disease [11,27].

The proportion of cases arising from a non-odontogenic source rose approximately threefold between the eras, from roughly one in eleven pre-pandemic patients to about one in four pandemic/post-pandemic patients. Although these counts are small and the difference is not statistically robust, several converging mechanisms could plausibly contribute to such a shift. First, the deferral of routine dental and primary care during lockdowns is likely to have been paralleled by the deferral of care for sore throat, tonsillar, and other oropharyngeal complaints, allowing a larger share of pharyngeal and tonsillar foci to progress to deep-neck necrotizing infection. Second, the post-pandemic period has been marked by a documented resurgence of invasive group A and other beta-hemolytic streptococcal disease, frequently in association with preceding or coexisting respiratory viral infection [11,27], which would be expected to increase the relative contribution of tonsillopharyngeal rather than odontogenic portals of entry. Third, altered community antibiotic prescribing during and after the pandemic may have changed both the spectrum of organisms reaching the deep neck and the likelihood that early infections were incompletely treated [33]. Finally, the immunonutritional shift discussed above may have lowered the threshold at which non-dental mucosal foci escalate to fascial necrosis. These explanations are necessarily speculative given that the absolute numbers involved amount to only one vs. four cases, and they are offered as hypotheses to be tested in larger multicenter series rather than as established causes.

The overall in-hospital mortality of 11.5% is at or below the lower end of the 10–40% range reported in the contemporary CNF literature [1,2,3,5]. This favorable outcome should be interpreted cautiously given the modest sample size but is consistent with the deliberate institutional approach refined at our center over more than a decade: early contrast-enhanced CT-based diagnosis, aggressive surgical source control through multiple cervical incisions with wide decompression and drainage of the deep neck fascial spaces and necrotic collections, early airway control with a low threshold for tracheotomy, prompt initiation of broad-spectrum empirical intravenous antibiotics, repeated open wound irrigation and surgical revision when indicated, and early ICU-level management with goal-directed hemodynamic and nutritional support. Our approach mirrors the operative philosophy advocated in the 2025 Chinese consensus statement on adult necrotizing fasciitis [36] and is comparable to the recent Ottawa post-pandemic experience, whose mortality of 12.5% lies close to ours [9]. Regardless of exact comparisons, the practical message is unambiguous: the surgical aggressiveness required in CNF, “more and earlier” rather than “less and wait”, remains the cornerstone of the outcome and arguably becomes even more critical in the pandemic/post-pandemic setting given the altered host phenotype documented here. Three practical clinical implications follow for the immediate post-pandemic landscape. First, clinicians should maintain a heightened threshold of suspicion for CNF in any patient presenting with disproportionate cervical swelling, systemic toxicity, or poor response to first-line antibiotics. The incidence has risen; presentation may be delayed, and host reserves are typically depleted. Particular attention should be directed toward mandibular odontogenic foci, which accounted for 100% of culprit teeth in our cohort. Second, the emergence of atypical Gram-negative and fungal organisms highlights the need for future studies evaluating whether empirical antimicrobial strategies should be adapted in selected CNF populations. Third, and perhaps most operationally useful, every CNF admission should receive a rapid bedside calculation of NPAR, CAR/PNI, and either PNI or albumin, none of which requires anything beyond the standard admission panel of complete blood count, CRP, and albumin. Whether a patient simultaneously exceeding the NPAR cut-off of ~2.8 and the CAR/PNI cut-off of ~0.28 should be flagged as being at combined inflammation–nutrition risk, and whether such flagging should prompt earlier nutritional support, a lower threshold for repeat surgical revision, and early ICU consultation, is a hypothesis that would require prospective evaluation before any clinical use.

The principal strengths of this work are the long pre-pandemic observation window of a full decade with consistent admission criteria and a standardized surgical protocol; the setting at a national tertiary referral center with broad catchment, which mitigates but does not eliminate selection bias; the formal Poisson-based incidence comparison including a sensitivity analysis; the most comprehensive composite biomarker panel yet applied to CNF, including the novel albumin-integrated ratios NPAR, CAR/PNI, SIRI/albumin and AISI/albumin; and the methodologically conservative microbiological classification that excludes anaerobes from the primary analysis because of non-uniform anaerobic culturing. Several limitations must be acknowledged. The sample size of 26 patients is modest, characteristic of this rare disease at a single institution and underpowered for individual outcomes with low baseline rates such as mortality; the design is intrinsically hypothesis-generating, and the exploratory logistic models and ROC cut-offs require external validation. The single-center design limits generalizability. Individual SARS-CoV-2 serological status and vaccination history were not systematically documented in the retrospective record, which prevents individual-level causal inference and means that the COVID-19 immune-dysregulation hypothesis is supported by temporal association and biological plausibility rather than direct exposure measurement. The Poisson IRR assumes a stable reference population across 2010–2026; any change in our catchment area (new hospitals, shifting referral patterns) could bias the estimate. We were unable to compute the LRINEC score because glucose, sodium, hemoglobin, and creatinine were not uniformly abstracted. Procalcitonin was available only in the later era and could not be used as a comparable marker. Histopathological confirmation was available only in the subset of cases where tissue samples were submitted, which is typical of retrospective surgical practice but should be reported precisely where institutional records allow. The exclusion of anaerobes from the primary microbiological analysis, while methodologically appropriate given non-uniform anaerobic culturing, may cause us to underestimate the true microbiological diversification. Finally, no formal adjustment for multiple testing was applied beyond the exploratory Benjamini–Hochberg FDR correction reported in Table 2; individual *p*-values should therefore be interpreted as directional rather than confirmatory.

Beyond the points above, several additional limitations constrain interpretation and should be read alongside them. Detailed host-vulnerability data were not uniformly available in the retrospective record: we lacked systematic documentation of immunosuppressive conditions or therapies (including HIV status, solid-organ or hematological transplantation, chronic corticosteroid or biologic therapy, and chronic liver disease), of body mass index and obesity, and of oncological history resolved by tumor type, stage, or active treatment (the four oncological patients comprised one multiple myeloma and three head-and-neck squamous cell carcinomas, of the hypopharynx, the floor of the mouth, and the lower lip, but their stage and active-treatment status at the time of CNF were not uniformly documented). We were therefore unable to formally stratify patients into immunocompetent and immunocompromised groups or to adjust for these recognized modifiers of necrotizing-infection risk and severity, and residual confounding by host immune status cannot be excluded. In addition, the interval from symptom onset and from hospital admission to the first surgical debridement was not consistently captured because inflammatory and nutritional markers such as CRP and albumin vary with the timing of sampling relative to disease evolution and intervention; differences in these intervals between eras could have contributed to the observed biomarker shift independently of any change in host biology. The microbiological comparison spans sixteen years, over which culture techniques and species-level identification, including the routine adoption of matrix-assisted laser desorption/ionization time-of-flight mass spectrometry, have improved; some of the apparent diversification, particularly the later detection of atypical and fastidious organisms, may therefore partly reflect enhanced laboratory identification rather than a purely biological change in the infecting flora. Finally, of the full composite biomarker panel, only the albumin-integrated indices NPAR and CAR/PNI retained significance after false-discovery-rate correction, and these two markers alone should be carried forward as the priority candidates for prospective evaluation.

## 5. Conclusions

At a Croatian national tertiary referral center, cervical necrotizing fasciitis became substantially more frequent in the pandemic/post-pandemic era (IRR = 2.58), with the rise accompanied by a coherent triple shift: a more comorbid host, a distinct hypoalbuminemic hyperinflammatory immunonutritional profile best captured by albumin-integrated composite biomarkers (NPAR, CAR/PNI, NAR, CAR), and a diversified microbiological landscape with loss of S. pyogenes, persistence of the S. anginosus/constellatus group, and emergence of atypical organisms. Despite this more adverse profile, in-hospital mortality remained at 11.5%, plausibly reflecting standardized, aggressive, surgical and airway-focused management. Pandemic-era CNF appears not simply more frequent but qualitatively altered, for which post-COVID-19 immune dysregulation provides a biologically plausible but, in this small retrospective cohort, still unproven upstream hypothesis. NPAR (AUC 0.867) and CAR/PNI (AUC 0.855) are promising but as yet unvalidated candidates for bedside risk stratification and require prospective multicenter external validation before any clinical use.

## Figures and Tables

**Table 1:** Demographic and clinical characteristics by era.

Variable	Pre-Pandemic (*n* = 11)	Pandemic/Post-Pandemic (*n* = 15)	*p*
Age (years), median (IQR)	39 (28–59)	49 (38–64)	0.287
Male sex, *n* (%)	8 (73%)	11 (73%)	1.000
Length of stay (days), median (IQR)	12 (9–24)	21 (16–29)	0.212
Odontogenic source, *n* (%)	10 (91%)	11 (73%)	0.356
Non-odontogenic (tonsillar/other), *n* (%)	1 (9%)	4 (27%)	0.356
Any comorbidity ≥ 1, *n* (%)	3 (27%)	10 (67%)	0.111
Diabetes mellitus, *n* (%)	1 (9%)	6 (40%)	0.178
Arterial hypertension, *n* (%)	1 (9%)	6 (40%)	0.178
Oncological disease, *n* (%)	3 (27%)	1 (7%)	0.279
Any complication, *n* (%)	3 (27%)	8 (53%)	0.246
Descending necrotizing mediastinitis, *n* (%)	2 (18%)	5 (33%)	0.658
Pneumonia, *n* (%)	0 (0%)	2 (13%)	0.492
Carotid rupture, *n* (%)	1 (9%)	0 (0%)	0.423
Osteonecrosis, *n* (%)	0 (0%)	1 (7%)	1.000
In-hospital mortality, *n* (%)	1 (9.1%)	2 (13.3%)	1.000

**Table 2:** Admission laboratory parameters and composite biomarkers by era.

Variable	Pre-Pandemic (*n* = 11)	Pand./Post-Pandemic (*n* = 15)	Cliff’s δ	*p* (q)
***Standard laboratory parameters***				
Leukocytes (×10^9^/L)	17.1 (12.8–20.0)	18.9 (15.2–23.0)	+0.23	0.337 (0.366)
Neutrophils (×10^9^/L)	11.60 (5.60–15.15)	16.80 (12.71–19.45)	+0.37	0.119 (0.172)
Lymphocytes (×10^9^/L)	1.20 (0.90–1.65)	0.77 (0.55–1.50)	−0.22	0.350 (0.366)
Monocytes (×10^9^/L)	0.60 (0.35–0.90)	0.72 (0.49–1.08)	+0.21	0.377 (0.377)
Platelets (×10^9^/L)	210 (161–224)	247 (193–302)	+0.35	0.146 (0.177)
CRP (mg/L)	295 (212–319)	346 (284–376)	+0.35	0.139 (0.177)
Albumin (g/L)	33.0 (30.0–35.5)	28.0 (23.0–31.0)	−0.58	0.013 (0.061)
**Procalcitonin (ng/mL)**	NA *	2.10 (0.76–11.36)	-	-
***Classical leukocyte-based indices***				
NLR	9.67 (4.27–13.50)	14.25 (9.34–33.47)	+0.41	0.087 (0.143)
PLR	210.0 (110.4–238.3)	266.7 (159.6–432.8)	+0.28	0.233 (0.267)
LMR	2.20 (1.42–2.38)	1.22 (0.83–2.22)	−0.38	0.108 (0.165)
SII	1661 (937–3065)	3278 (1894–7533)	+0.36	0.132 (0.177)
AISI	1089 (506–1998)	3241 (1288–5613)	+0.48	0.043 (0.090)
SIRI	5.09 (3.04–7.65)	14.48 (5.89–19.76)	+0.45	0.055 (0.105)
dNLR	4.30 (1.62–7.05)	7.57 (5.45–11.19)	+0.42	0.078 (0.137)
***Albumin-integrated composite markers***				
CAR	8.20 (7.38–9.81)	12.57 (9.34–17.01)	+0.61	0.009 (0.054)
NAR	0.350 (0.189–0.415)	0.568 (0.496–0.680)	+0.61	0.009 (0.054)
NPAR	2.41 (2.00–2.47)	3.03 (2.71–3.93)	+0.73	**0.002 (0.030)**
PNI	40.0 (37.2–42.5)	33.5 (28.2–37.1)	−0.49	0.038 (0.087)
CALLY	1.71 (1.18–2.08)	0.75 (0.45–1.31)	−0.53	0.026 (0.066)
CAR/PNI	0.198 (0.183–0.252)	0.400 (0.261–0.489)	+0.71	**0.003 (0.030)**
SIRI/albumin	0.159 (0.087–0.219)	0.569 (0.206–0.787)	+0.56	0.017 (0.065)
AISI/albumin	28.7 (15.8–58.4)	120.9 (48.9–239.3)	+0.54	0.022 (0.066)
mGPS (0–2)	2.0 (1.0–2.0)	2.0 (2.0–2.0)	+0.39	0.025 (0.066)

Cliff’s δ is oriented as pandemic/post-pandemic minus pre-pandemic; |δ| ≥ 0.33 indicates a medium effect, |δ| ≥ 0.47 indicates a large effect. BH q = Benjamini–Hochberg false-discovery-rate-adjusted q value across the continuous biomarker panel (*n* = 22 tests). * Procalcitonin entered routine use only in 2020; no pre-pandemic values were available, precluding between-era comparison (available in 14/15 later-era patients). Abbreviations: NLR, neutrophil-to-lymphocyte ratio; PLR, platelet-to-lymphocyte ratio; LMR, lymphocyte-to-monocyte ratio; SII, systemic immune–inflammation index; AISI, aggregate index of systemic inflammation; SIRI, systemic inflammation response index; dNLR, derived neutrophil-to-lymphocyte ratio; CAR, C-reactive-protein-to-albumin ratio; NAR, neutrophil-to-albumin ratio; NPAR, neutrophil-percentage-to-albumin ratio; PNI, prognostic nutritional index; CALLY, C-reactive-protein–albumin–lymphocyte index; CAR/PNI, SIRI/albumin and AISI/albumin, the corresponding composite ratios; mGPS, modified Glasgow Prognostic Score.

**Table 3:** ROC performance of leading biomarkers for era discrimination.

Marker	AUC (95% CI)	Cut-off	Direction	Sens.	Spec.	PPV/NPV
NPAR	0.867 (0.697–0.994)	2.789	higher post	0.73	1.00	1.00/0.73
CAR/PNI	0.855 (0.691–0.976)	0.283	higher post	0.73	1.00	1.00/0.73
NAR	0.806 (0.606–0.970)	0.471	higher post	0.80	0.82	0.86/0.75
CAR	0.806 (0.624–0.945)	12.567	higher post	0.53	1.00	1.00/0.61
Albumin (inverse)	0.791 (0.600–0.949)	28.000	lower post	0.67	0.82	0.83/0.64
SIRI/Albumin	0.782 (0.576–0.945)	0.186	higher post	0.87	0.73	0.81/0.80
AISI/Albumin	0.770 (0.576–0.939)	120.910	higher post	0.53	1.00	1.00/0.61
CALLY (inverse)	0.764 (0.558–0.927)	1.362	lower post	0.80	0.64	0.75/0.70
PNI (inverse)	0.745 (0.536–0.933)	36.150	lower post	0.73	0.82	0.85/0.69
AISI	0.739 (0.539–0.921)	1246.560	higher post	0.87	0.64	0.76/0.78

Cut-offs selected by Youden’s index on the single-variable ROC. Direction indicates whether higher or lower values flag the pandemic/post-pandemic era. AUC = area under the receiver operating characteristic curve; 95% CI by 2000-iteration stratified bootstrap. Abbreviations: AUC, area under the receiver operating characteristic curve; CI, confidence interval; Sens., sensitivity; Spec., specificity; PPV, positive predictive value; NPV, negative predictive value; NPAR, neutrophil-percentage-to-albumin ratio; CAR, C-reactive-protein-to-albumin ratio; NAR, neutrophil-to-albumin ratio; CAR/PNI, SIRI/albumin and AISI/albumin, composite ratios; CALLY, C-reactive-protein–albumin–lymphocyte index; PNI, prognostic nutritional index; AISI, aggregate index of systemic inflammation.

**Table 4:** Microbiological categories by era.

Category	Pre-Pandemic (*n* = 11)	Pand./Post-Pandemic (*n* = 15)	*p*
***Streptococci***—total	9 (82%)	5 (33%)	0.021
*S. pyogenes* (Group A)	1 (9%)	0 (0%)	0.423
***S. anginosus*/*constellatus group***	3 (27%)	5 (33%)	1.000
Other ***viridans*** (***S. mitis*, *S. oralis, S. thermophilus***)	1 (9%)	1 (7%)	1.000
***Staphylococci***	1 (9%)	1 (7%)	1.000
Monomicrobial streptococcal infection	6 (55%)	2 (13%)	0.038
Polymicrobial infection (≥ 2 genera)	2 (18%)	4 (27%)	1.000
Atypical organisms (***A. baumannii, Candida, C. striatum***) *	0 (0%)	3 (20%)	0.238
Unidentified/negative culture	2 (18%)	5 (33%)	0.658

* Atypical = *Acinetobacter baumannii, Candida albicans, Corynebacterium striatum* (uncommon in typical odontogenic/cervicofacial infection). Anaerobes are not compared as a separate category because systematic anaerobic culturing was not performed uniformly across the entire observation period; when identified, they contributed to the polymicrobial category. Species-level streptococcal interpretation is limited by evolving laboratory diagnostics: older records frequently reported “Streptococcus species” without species-level detail.

## Data Availability

All data generated or analyzed during this study are included in the published article.

## Supplementary Materials

The following supporting information can be downloaded at https://www.mdpi.com/article/10.3390/jcm15145350/s1; Table S1. Composite biomarker formulae and biological rationale.

## Abbreviations

The following abbreviations are used in this manuscript:

AISI	Aggregate Index of Systemic Inflammation
AUC	Area Under the Receiver Operating Characteristic Curve
BH-FDR	Benjamini–Hochberg False-Discovery Rate
CALLY	CRP–Albumin–Lymphocyte Index
CAR	C-Reactive Protein to Albumin Ratio
CNF	Cervical Necrotizing Fasciitis
COVID-19	Coronavirus Disease 2019
CRP	C-Reactive Protein
DM	Diabetes Mellitus
dNLR	Derived Neutrophil-to-Lymphocyte Ratio
FDI	Fédération Dentaire Internationale (Tooth Numbering System)
GAS	Group A Streptococcus
LMR	Lymphocyte-to-Monocyte Ratio
LOOCV	Leave-One-Out Cross-Validation
LRINEC	Laboratory Risk Indicator for Necrotizing Fasciitis
MDR	Multidrug-Resistant
mGPS	Modified Glasgow Prognostic Score
NAR	Neutrophil-to-Albumin Ratio
NLR	Neutrophil-to-Lymphocyte Ratio
NPAR	Neutrophil Percentage-to-Albumin Ratio
NSTI	Necrotizing Soft Tissue Infection
PCT	Procalcitonin
PLR	Platelet-to-Lymphocyte Ratio
PNI	Prognostic Nutritional Index
SAG	Streptococcus anginosus Group
SARS-CoV-2	Severe Acute Respiratory Syndrome Coronavirus 2
SII	Systemic Immune–Inflammation Index
SIRI	Systemic Inflammation Response Index
