# Supplementary material for: Cervical Necrotizing Fasciitis Across the COVID-19 Pandemic: A Single-Center Exploratory Retrospective Cohort Study
Source: J Clin Med. 2026 Jul 8;15(14):5350. doi: 10.3390/jcm15145350 (PMC13410499; doi:10.3390/jcm15145350)
Supplement: Supplementary file 1 [file jcm-15-05350-s001.zip › jcm-4371452-supplementary.pdf]

Supplementary Table S1. Composite biomarker formulae and biological rationale.

All biomarkers were calculated from admission laboratory values. Albumin is expressed in g/L; absolute leukocyte, neutrophil, lymphocyte, monocyte and platelet counts in $\times 10^9$/L; and C-reactive protein (CRP) in mg/L. Classical leukocyte-based indices summarise circulating inflammatory-cell distributions, whereas the albumin-integrated composites additionally incorporate serum albumin (or the prognostic nutritional index) to capture the host's nutritional and vascular-reserve status.

**Biomarker**	**Formula**	**Primary biological axis**
NLR	neutrophils ÷ lymphocytes	Neutrophil-dominant innate activation vs lymphocyte suppression
PLR	platelets ÷ lymphocytes	Platelet/thrombo-inflammatory activation vs lymphocyte suppression
LMR	lymphocytes ÷ monocytes	Adaptive vs monocytic compartment balance
SII	(platelets × neutrophils) ÷ lymphocytes	Aggregate systemic immune-inflammation
AISI	(neutrophils × monocytes × platelets) ÷ lymphocytes	Aggregate index of systemic inflammation
SIRI	(neutrophils × monocytes) ÷ lymphocytes	Systemic inflammation response index
dNLR	neutrophils ÷ (leukocytes − neutrophils)	Derived neutrophil-to-lymphocyte ratio
CAR	CRP ÷ albumin	Acute-phase inflammation relative to visceral protein reserve
NAR	neutrophils ÷ albumin	Neutrophilic inflammation relative to nutritional reserve
NPAR	100 × (neutrophils ÷ leukocytes) ÷ albumin	Neutrophil percentage relative to albumin (inflammation–nutrition)
PNI	albumin + 5 × lymphocytes (Onodera)	Prognostic nutritional / immunonutritional reserve
CALLY	(albumin × lymphocytes) ÷ CRP × 10	Combined nutrition, immunity and inflammation
CAR/PNI	CAR ÷ PNI	Inflammation normalised to immunonutritional reserve
SIRI/albumin	SIRI ÷ albumin	Cellular inflammation normalised to nutritional reserve
AISI/albumin	AISI ÷ albumin	Aggregate inflammation normalised to nutritional reserve
CRP × NLR	CRP × NLR	Multiplicative humoral × cellular inflammatory composite
mGPS	0 (CRP ≤ 10 mg/L and albumin ≥ 35 g/L); 1 (CRP > 10 and albumin ≥ 35); 2 (CRP > 10 and albumin < 35)	Modified Glasgow Prognostic Score (inflammation + hypoalbuminemia)

Abbreviations: CRP, C-reactive protein; NLR, neutrophil-to-lymphocyte ratio; PLR, platelet-to-lymphocyte ratio; LMR, lymphocyte-to-monocyte ratio; SII, systemic immune-inflammation index; AISI, aggregate index of systemic inflammation; SIRI, systemic inflammation response index; dNLR, derived neutrophil-to-lymphocyte ratio; CAR, C-reactive-protein-to-albumin ratio; NAR, neutrophil-to-albumin ratio; NPAR, neutrophil-percentage-to-albumin ratio; PNI, prognostic nutritional index; CALLY, C-reactive-protein–albumin–lymphocyte index; mGPS, modified Glasgow Prognostic Score. The albumin-integrated composite indices were pre-specified rather than data-driven.
